# Are Individual and Community Acceptance and Witnessing of Intimate Partner Violence Related to Its Occurrence? Multilevel Structural Equation Model

**DOI:** 10.1371/journal.pone.0027738

**Published:** 2011-12-14

**Authors:** Olalekan A. Uthman, Tahereh Moradi, Stephen Lawoko

**Affiliations:** 1 Division of Social Medicine, Department of Public Health Sciences, Karolinska Institutet, Stockholm, Sweden; 2 Division of Epidemiology, Department of Environmental Medicine, Karolinska Institutet, Stockholm, Sweden; Tulane University, United States of America

## Abstract

**Background:**

Intimate partner violence against women (IPVAW) is a serious and widespread problem worldwide. Much of the research on IPVAW focused on individual-level factors and attention has been paid to the contextual factors. The aim of this study was to develop and test a model of individual- and community-level factors associated with IPVAW.

**Methods and Findings:**

We conducted a (multivariate) multilevel structural equation analysis on 8731 couples nested within 883 communities in Nigerian Demographic and Health Survey 2008. Variables included in the model were derived from respondents' answers to the experience of IPVAW, attitudes towards wife beating and witnessing physical violence in childhood. We found that women that witnessed physical violence were more likely to have tolerant attitudes towards IPVAW and women with tolerant attitudes were more likely to have reported spousal IPVAW abuse. Women with husbands with tolerant attitudes towards IPVAW were more likely to have reported spousal abuse. We found that an increasing proportion of women in the community with tolerant attitudes was significantly positively associated with spousal sexual and emotional abuse, but not significantly associated with spousal physical abuse. In addition, we found that an increasing proportion of men in the community with tolerant attitudes and an increasing proportion of women who had witnessed physical violence in the community was significantly positively associated with spousal physical abuse, but not significantly associated with spousal sexual and emotional abuse. There was a positive correlation between all three types of IPVAW at individual- and community-level.

**Conclusions:**

We found that community tolerant attitudes context in which people live is associated with exposure to IPVAW even after taking into account individual tolerant attitudes. Public health interventions designed to reduce IPVAW must address people and the communities in which they live in order to be successful.

## Introduction

Intimate partner violence against women (IPVAW) is defined as threatened, attempted, or completed physical or sexual or emotional abuse [1]. IPVAW can be committed by a spouse, an ex-spouse, a current or former boyfriend or girlfriend, or a dating partner [1]. Intimate partner violence against women are serious and widespread problems worldwide [2]. IPVAW has been linked to numerous immediate and long-term health consequences, including but not limited to physical injury, unwanted pregnancy, abortion, gynaecological complications, sexually transmitted infections (including human immunodeficiency virus), posttraumatic stress disorder and depression, among others [2]. The World Health Organization (WHO) Multi-country study on women's health and domestic violence against women indicated that 15–71% of women experience physical and/or sexual violence by an intimate partner at some point in their lives [3].

Numerous studies have found that demographic, social, empowerment and behavioural factors may be associated with vulnerability to IPVAW [2]. Of all these factors, attitudes towards IPVAW have been found to be one of the strongest predictors of exposure to IPVAW. Although research has paid some attention to the tolerant attitudes towards IPVAW at individual-level, almost no attention has been paid to the community-level tolerant attitudes towards IPVAW. To the best of our knowledge, there has been no multilevel study performed to date that examined the separate and independent effect community-level tolerant attitudes towards IPVAW on women experience of IPVAW in sub-Saharan Africa. An understanding of determinants of IPVAW beyond individual characteristics (i.e. at community-level) is necessary for the development of appropriate intervention of benefit to the community at large. Communities are important in shaping of disparities in health, as they shape individual opportunity and expose residents to multiple risks and resources over the life course [4], [5]. Focusing only at one level—either the micro individual level or the macro scale of contexts—generates conceptual and practical problems [6]. Therefore, to expand our understanding of the risk factors associated with IPVAW, we considered an additional risk factor, the characteristics of the communities in which women live.

The intergenerational recycling of IPVAW has been an area of fundamental debate over the past decades. Data has indicated that women who experienced or witnessed abuse during childhood are more likely to be victims of abuse in adulthood. Paradoxically, men who had experienced or witnessed abuse in childhood may become perpetrators of abuse in adulthood [7].

The mechanism linking the witnessing of abuse in childhood to the exposure of abuse is not yet well established. It is reasonable to argue that attitudes towards abuse may be a pathway linking childhood witnessing to adulthood behaviour (i.e. as postulated in the social learning theory described below). Thus the association between witnessing of physical violence in childhood, attitudes towards abuse and exposure to IPVAW warrants further understanding.

### Conceptual model

We developed a working conceptual framework to explore at a high level how community-level tolerant attitudes towards IPVAW may influence women experience of IPVAW. To operationalize the framework, we borrow from social learning theory [8] and ecological framework [9]. Ecological theory has been used widely by family violence researchers to understand partner abuse [9], [10]. This framework conceptualizes IPVAW as a multifaceted phenomenon grounded on interaction between individual, family, community, and societal factors. The ecology theory takes into account the different levels of societal organization and their role in influencing attitudes towards IPVAW. An individual resides in a household unit, which in turn is situated within a community, which will operate under the policies of a state or national government. Each level within the societal structure has the potential to influence individual attitudes towards IPVAW. Based on models initially developed by Bandura [11], [12], social learning theorists hypothesized that IPVAW is initially acquired through modelling during childhood. The theory proposes that methods for settling family conflicts are often learned during childhood by observing parental and peer relationships [8], [13]. Victims and perpetrators of partner abuse are thought to have either witnessed abuse as children, resulting in the development of tolerance or acceptance of violence within the family [14].

In this article, we take advantage of a unique couple data set from Nigerian Demographic and Health Survey (DHS) 2008. This data set permits to develop and test a (multivariate) multilevel structural equation model of factors associated with IPVAW that includes individual-level characteristics along with contextual characteristics at community level. Specifically, we focused on the effects of individual- and community-level tolerant attitudes and witnessing physical violence in childhood. The model included the following hypotheses:

- *Hypothesis 1*: women who had witnessed physical violence in childhood were more likely to have tolerant attitudes towards IPVAW (represented by path coefficient).
- *Hypothesis 2*: women with tolerant attitudes towards IPVAW were more likely to experience spousal physical, sexual and emotional abuse.
- *Hypothesis 3*: women with husband with tolerant attitudes towards IPVAW were more likely to experience spousal physical, sexual and emotional abuse.
- *Hypothesis 4*: Increasing tolerance of IPVAW among women at the community level will be positively associated with exposure to spousal physical, sexual and emotional abuse.
- *Hypothesis 5*: Increasing tolerance of IPVAW among men at the community level will be positively associated with exposure to spousal physical, sexual and emotional abuse.
- *Hypothesis 6*: Increasing ratio of women who had witnessed IPVAW in childhood in the community will be positively associated with exposure to spousal physical, sexual and emotional abuse.
- *Hypothesis 7*: All three forms of IPVAW are likely to co-vary at both individual-level (physical vs. emotional, physical vs. sexual and sexual vs. emotional) and community-level (physical vs. emotional, physical vs. sexual and sexual vs. emotional).
- *Hypothesis 8*: Husband and wife tolerant attitudes towards IPVAW are likely to co-vary at both individual-level and community-level, such that women with tolerant attitudes towards IPVAW were more likely to have husbands with tolerant attitudes towards IPVAW.

To assess for mediator, unidirectional or non-recursive associations, the following hypotheses were tested:

- *Hypothesis 9*: Women tolerant attitudes towards IPVAW will mediate association between witnessing IPVAW and exposure to IPVAW.
- *Hypothesis 10*: The association will be observed from tolerant attitudes to exposure to IPVAW and also exposure to IPVAW and tolerant attitudes.

## Methods

### Setting

Nigeria is located in western Africa on the Gulf of Guinea and has a total area of 923,768 kilometer squared (km^2^), making it the world's 32nd-largest country (after Tanzania). Nigeria is the most populous country in Africa. The United Nations estimates that the population in 2004 was at 131,530,000, with the population distributed as 48.3% urban and 51.7% rural and population density at 139 people per km^2^. Nigeria has more than 250 ethnic groups, with varying languages and customs, creating a country of rich ethnic diversity. The largest ethnic groups are the Fulani/Hausa, Yoruba, Igbo, accounting for 68% of population, while the Edo, Ijaw, Kanuri, Ibibio, Ebira Nupe and Tiv comprise 27%; other minorities make up the remaining 5%. The middle belt of Nigeria is known for its diversity of ethnic groups, including the Pyem, Goemai, and Kofyar.

### Study design

Cross-sectional and population-based study using data from the 2008 Nigerian Demographic and Health survey (NDHS).

### Sampling technique

Methods used in the NDHS have been published elsewhere [15]. Briefly, the survey used a two-stage cluster sampling technique. The country was stratified into 36 States and the Federal Capital Territory (FCT), Abuja. Administratively, Nigeria is divided into States. Each State is subdivided into local government areas (LGAs), and each LGA is divided into localities. In addition to these administrative units, during the 2006 Population Census, each locality was subdivided into convenient areas called census enumeration areas (EAs). The primary sampling unit (PSU), referred to as a cluster for the 2008 NDHS, is defined on the basis of EAs from the 2006 EA census frame. The 2008 NDHS sample was selected using a stratified two-stage cluster design consisting of 886 clusters. The first stage involved selecting 886 clusters (primary sampling units) with a probability proportional to the size, the size being the number of households in the cluster. The second stage involved the systematic sampling of households from the selected clusters. A total of 36.298 households was selected for the 2008 NDHS survey and of these 34,644 were occupied.

Of the 34,644 households found, 34,070 were successfully interviewed, yielding a response rate of 98 per cent. In the interviewed households, a total of 34, 596 women were identified to be eligible for the individual interview, and 97 per cent of them were successfully interviewed. For men, 16,722 were identified as eligible in half the households, and 93 per cent of them were successfully interviewed. One randomly selected woman age 15–49 per household was selected for domestic violence module [16]; however, only women who were ever-married or have ever cohabited were eligible for the questions in the module related to spousal violence.

### Data collection

Data collection procedures have been published elsewhere [15]. Briefly, data were collected by visiting households and conducting face-to-face interviews to obtain information on demographic characteristics, wealth, anthropometry, female genital cutting, HIV knowledge, sexual behaviour, and domestic violence.

### Ethical consideration

This study was based on an analysis of existing survey data with all identifier information removed. The survey was approved by the Ethics Committee of the ICF Macro at Calverton in the USA and by the National Ethics Committee in the Ministry of Health in Nigeria. Written consent was obtained from all respondents and all information was collected confidentially.

### Variables

This sample was a subset of the couple's file and includes only currently married or cohabiting women age 20–44 who were administered the domestic violence module and completed the questions related to spousal violence and whose husbands/partners were interviewed with the men's questionnaire. This subsample was used for analyses in which couples are the relevant unit of analysis and that involve the questions on IPVAW.

#### Individual-level (couples)

Two latent variables (IPVAW and acceptance of wife beating) and one measured variable were included at individual-level. The latent variables are variables that are not directly observed but are rather inferred (through a mathematical model) from other variables that are observed (directly measured).

#### IPVAW

IPVAW (spousal physical, sexual and emotional abuse) were assessed using a modified and previously validated version of the Conflict Tactic Scale [17], where IPVAW was defined as exposure to one or several of the following experiences perpetrated by a husband/partner ever. IPVAW Six variables were used to measure physical abuse: spouse ever pushed, shook or threw something; spouse ever slapped; spouse ever punched with fist or something harmful; spouse ever kicked or dragged; spouse ever tried to strangle or burn; and spouse ever threatened with knife/gun or other weapon. Two variables were used to measure sexual abuse: forced sexual intercourse and other sexual act when undesired. Three variables were used to measure emotional abuse: spouse ever humiliated her in public; spouse ever threatened her with harm; and spouse ever insult or make feel bad.

#### Acceptance of wife beating

Husband and wife acceptance of wife beating (tolerant attitudes towards IPVAW) was constructed from five variables on whether husband is justified in hitting or beating his wife if she transgressed established gender roles? The five scenarios for justifying wife beating were: (1) “if wife burns the food,” (2) “if wife argues with the husband,” (3) “if wife goes out without informing the husband,” (4) “if wife neglects the children,” and (5) “if the wife refuses to have sexual relations with the husband”.

#### Witnessed physical violence in childhood

Whether respondents witnessed physical violence or not during their childhood were assessed by inquiring whether their father ever beat her mother?

#### Community-level

We used the term community to describe clustering within the same geographical living environment. Communities were based on sharing a common primary sample unit (PSU) within the DHS data. The most recent census was used to identify PSU. Census enumeration blocks and villages were used to identify PSU in urban and rural areas respectively. Each PSU must contain at least 50 households. Villages with less than 50 households were joined with adjoining neighbouring village. Villages with more than 500 households were classified as one PSU, although it will be segmented, with a sub-sample of segments being selected for household listing and interviewing. The unit of analysis was chosen for two reasons. First, PSU is the most consistent measure of community across all the surveys [18], and thus the most appropriate identifier of community for this cross-region comparison. Second, it has been shown that for most of the DHS conducted, the sample size per cluster met the optimum size with a tolerable precision loss [19] (The bias introduced by using cluster averages based on about 25 women as a proxy for the PSU population averages is very small – only about 4% [20]. Percentage of respondents with tolerant attitudes towards IPVAW and percentage of respondents that witnessed physical violence in childhood were derived from the DHS at community-level.

### Statistical analyses

**Figure 1:**
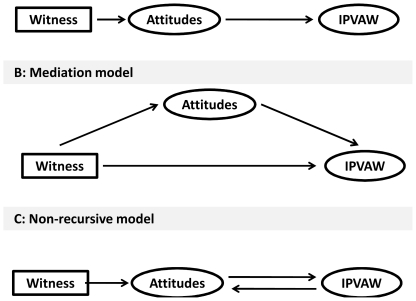
Alternative hypotheses for the association between tolerant attitudes towards IPVAW, witnessing IPVAW and experience of IPVAW.

We adopted the two-step approach proposed by Anderson and Gerbing [21], [22] for analysing the postulated model, where a confirmatory measurement model was specified prior to the simultaneous estimation of the measurement and the structural model. Figure 1 show three hypothesized association between witnessing physical violence, women tolerant attitudes and exposure to IPVAW. Prior to testing the final multilevel structural equation model, we examined three alternative models, whether unidirectional, moderation and reciprocal models would better fit the data. Unidirectional model assumes that women who had witnessed physical violence will develop tolerant attitudes towards and their tolerant attitudes will be associated with experience of IPVAW (Figure 1A). Moderating model assumes that women tolerant attitudes towards IPVAW will mediate association between witnessing IPVAW and exposure to IPVAW (Figure 1B). Reciprocal model assumes that association will be observed from tolerant attitudes to exposure to IPVAW and also exposure to IPVAW and tolerant attitudes (Figure 1C). These theoretical models were then tested and revised until a theoretically meaningful and statistically acceptable model was found.

### Model fit diagnosis

We conducted model testing with the Mplus analytic program [23]. We evaluated model fit by examining the following fit indicators, using criteria suggested by Hu and Bentler [24]. These include examination of chi-square statistics, a comparative fit index (CFI), a Tucker-Lewis index (TLI) and a root mean square error of approximation (RMSEA). The chi-square statistics indicate the corresponding between the proposed model and data. The RMSEA is a measure of the error of approximation between hypothesized model-implied covariance matrix in the sample and the population covariance matrix. The CFI assessed the improvement in fit of the hypothesized model compared with a baseline model (i.e. null model), when covariances among the population are assumed to be zero. The TLI corrects for model complexity, favouring parsimonious models over more complex ones. Values for the RMSEA ranging from 0 to 0.05 and for CFI and TLI above 0.90 and 0.95, respectively, represent acceptable fit of the model.

## Results

### Characteristics of the couples

The study analysed 8731 couples living in 883 communities in Nigerian DHS 2008. Table 1 shows the summary characteristics of the respondents. About 10% of the women reported spousal physical abuse and 14% reported emotional abuse by their partner. Only 2% reported spousal sexual abuse. Among couples, about 37% of husband and wife did not justify wife beating. About 13% of husband alone justify wife beating, while 32% of wives alone justified wife beating for transgressing certain gender roles. Nearly one-fifth (17%) of both husband and wife justified wife beating. Less than one-tenth (8%) of the respondents witnessed physical violence in their childhood.

**Table 1:** Summary statistics of couples, Nigerian DHS 2008.

Variable	Percentage
**Individual-level (n = 8731)**	
Intimate partner violence	
Physical abuse	10.4
Sexual abuse	2.3
Emotional abuse	14.3
Couple attitudes	
Neither	36.9
Husband alone	13.4
Wife alone	32.0
Both	17.7
Women witnessed IPVAW	7.7
**Community-level (n = 883)**	Mean (SD)
Intimate partner violence	
Physical abuse	12.8 (17.0)
Sexual abuse	2.6 (7.4)
Emotional abuse	15.4 (17.1)
Couple attitudes	
Neither	39.6 (28.7)
Husband alone	13.4 (16.3)
Wife alone	29.6 (23.8)
Both	17.4 (21.5)
Women witnessed IPVAW	10.1 (16.7)

### Measurement model

**Figure 2:**
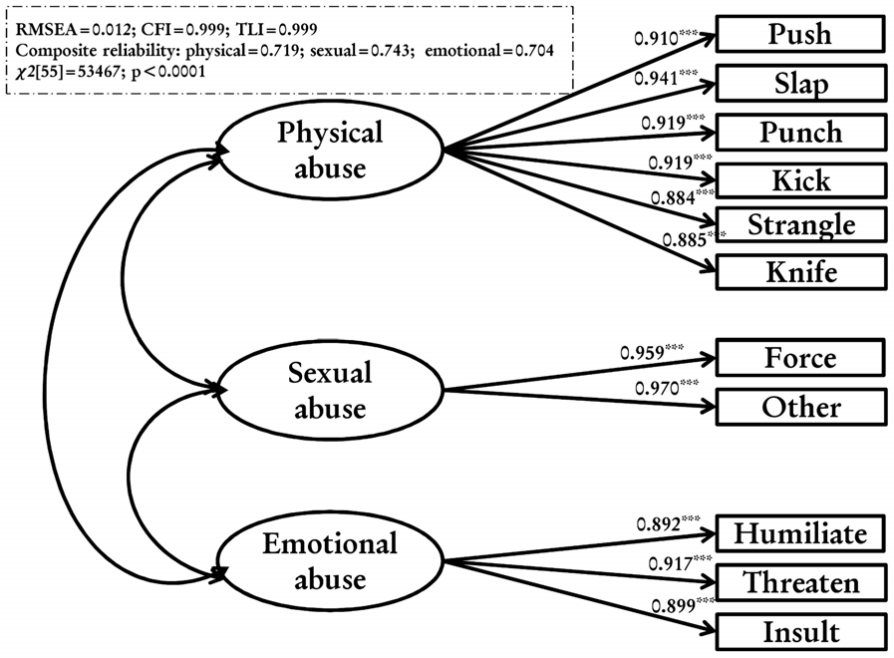
Item analysis, goodness-of-fit, reliability and validity assessment of the experience of IPVAW measurement model.

**Figure 3:**
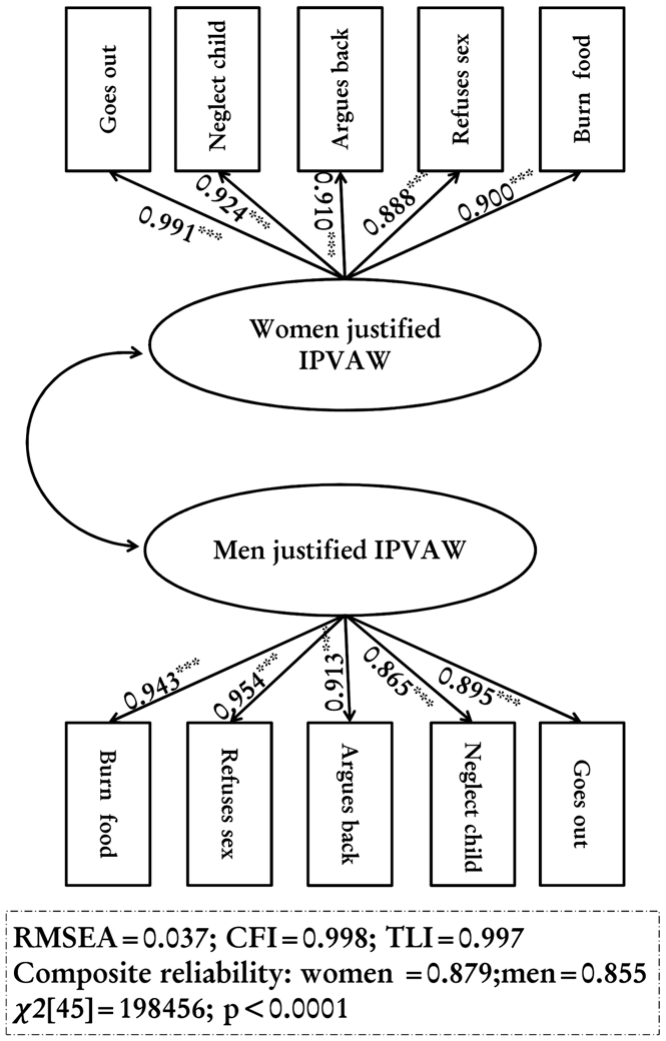
Item analysis, goodness-of-fit, reliability and validity assessment of the couple's tolerant attitudes towards IPVAW measurement model.

Figure 2 shows the IPVAW measurement model, which has acceptable practical fit indices. All sub-constructs had factor loadings above 0.8. The composite reliability shows the excellent consistency of the indicators in measuring the three latent variables: spousal physical, sexual and emotional abuse. The validity of the constructs is also supported by the χ2 difference test and the variance extracted test. Combined, these findings support the reliability and validity of the three constructs and their indicators and indicated that items loaded on appropriate latent variables. Figure 3 shows the results of tolerant attitudes towards IPVAW measurement model, which has acceptable practical fit indices. All sub-constructs had factor loadings above 0.8. The composite reliability shows the excellent consistency of the indicators in measuring both latent variables: husband and wife tolerant attitudes towards IPVAW. The validity of the constructs is also supported by the χ2 difference test and the variance extracted test. Combined, these findings support the reliability and validity of the two constructs and their indicators and indicated that items loaded on appropriate latent variables.

### Model selection

The hypothesis that there is unidirectional association between witnessing IPVAW, tolerant attitudes and exposure to IPVAW was supported. The hypothesis suggesting that tolerant attitudes mediate association between witnessing IPVAW and experience of IPVAW was not supported. Witnessing IPVAW was not indirectly associated with exposure to IPVAW via tolerant attitudes. The indirect effect was zero. Similarly, the hypothesis suggesting that there is reciprocal association between tolerant attitudes and exposure to IPVAW was not supported.

### Final model

**Figure 4:**
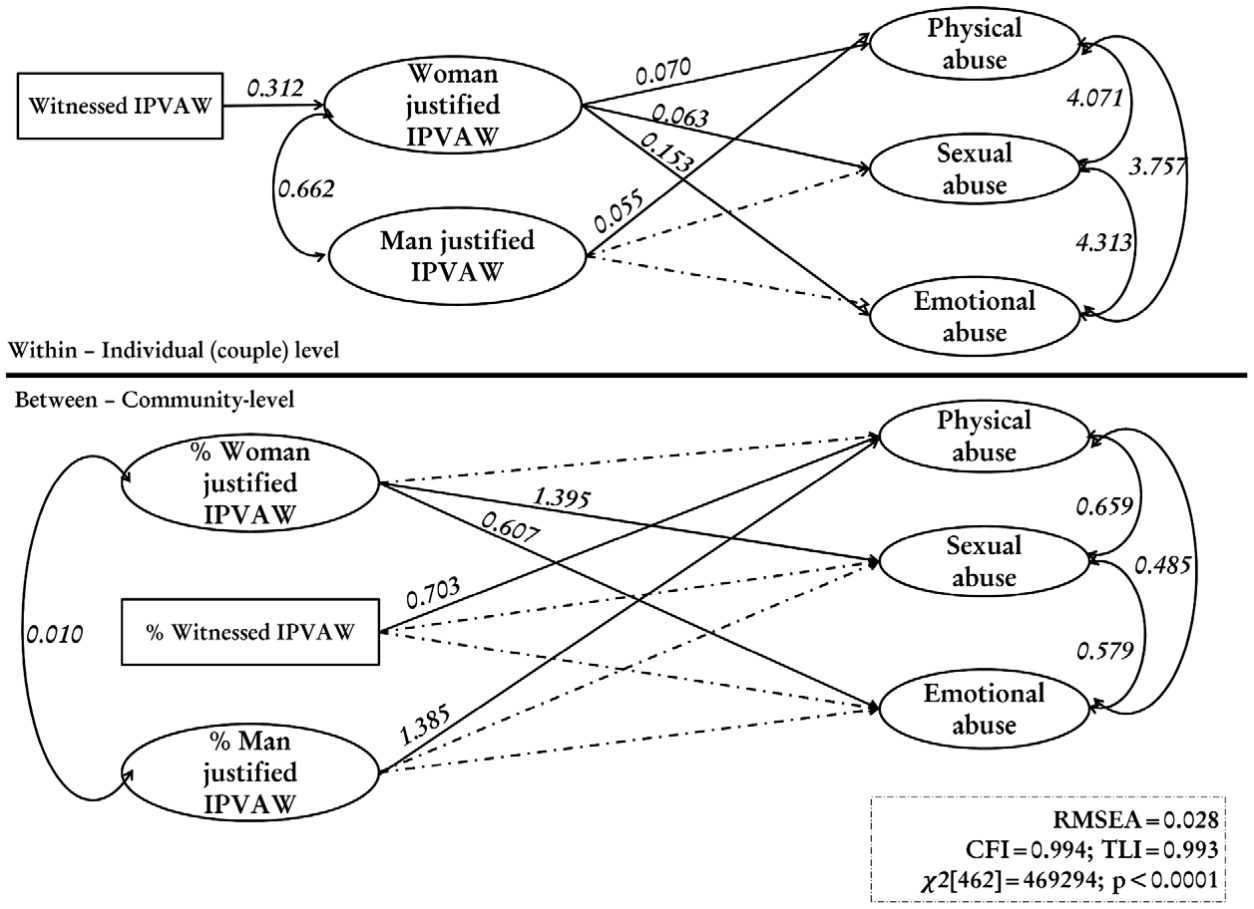
Final model.

The results of the final model are also presented in Figure 4. Only the paths that are statistically significant are shown. Standardized path coefficients appear on single-headed arrows. Correlations of the residual terms appear on curved double-headed arrows. According to goodness of fit indices, the final model provided a good fit to the data (x2(df = 462) = 469294, p<0.0001, RMSEA = 0.028, CFI = 0.994, TLI = 0.0993). As shown in Figure 4, the final model revealed that those women that witnessed IPVAW were more likely to have tolerant attitudes towards IPVAW (regression coefficient [β] = 0.312, p<0.001). Women with tolerant attitudes were more likely to have reported spousal physical (β = 0.070, p<0.001), sexual (β = 0.153, p<0.001) and emotional (β = 0.063, p<0.001) abuse. However, women with husband with tolerant attitudes towards IPVAW were more likely to have reported spousal physical abuse (β = 0.055, p = 0.034). The association between husband tolerant attitudes, spousal sexual and emotional abuse were not significant.

At community level, increasing women with tolerant attitudes towards IPVAW was positively associated with spousal sexual (β = 1.395, p = 0.010) and emotional abuse (β = 0.607, p = 0.007), but not physical abuse. Increasing men with tolerant attitudes towards IPVAW in the community was positively associated only spousal physical abuse (β = 0.703, p = 0.026), but not spousal sexual and emotional. Similarly, increasing women who had witnessed IPVAW in the community was positively associated only spousal physical abuse (β = 1.385, p = 0.004), but not spousal sexual and emotional.

There was positive correlation between all three types of IPVAW at both individual-level (physical vs. emotional [β = 3.757, p<0.001]; physical vs. sexual [β = 4.071, p<0.001]; and sexual vs. emotional [β = 4.313, p<0.001]) and community-level (physical vs. emotional [β = 0.485, p = 0.001]; physical vs. sexual [β = 0.659, p = 0.014]; and sexual vs. emotional [β = 0.579, p = 0.008]). For example, women who had experience physical abuse were also more likely to have experience emotional abuse, and vice versa. Similarly, communities that experienced high spousal physical violence were also more likely to have experienced high spousal sexual abuse and vice versa. In addition, there was positive correlation between husband and wife tolerant attitudes at both individual-level (β = 0.662, p<0.001) and community level (β = 0.010, p<0.001), such that women with tolerant attitudes were more likely to have husbands with tolerant attitudes too. However, community-level correlations between husband and wife tolerant attitudes were relatively less pronounced.

## Discussion

### Main findings

This article develops social learning and ecological theories to explore association between individual-, community-tolerant attitudes towards IPVAW and exposure to IPVAW. The key findings of this study are as follows. Firstly, we found that women that witnessed IPVAW were more likely to have tolerant attitudes towards IPVAW and women with tolerant attitudes were more likely to have reported spousal physical, sexual and emotional abuse. This is consistent with previous studies that found that attitude towards IPVAW is one of the most important factors associated with IPVAW [25], [26], [27]. In addition, we found support for the hypothesis that women with partner with tolerant attitudes towards IPVAW were more likely to have reported spousal abuse. In addition, women with tolerant attitudes were more likely to have partners with tolerant attitudes too. The hypothesis that “women who endorse cultural beliefs about husbands' right to use violence to control wives' behaviour will be more likely to experience spousal abuse” is based on assumption that women who adhere to more traditional notions of husband's rights and privileges are more likely to be married to men who raised in families in which traditional gender roles were encouraged [27]. Our alternative hypothesis that tolerant attitudes mediate association between witnessing IPVAW and exposure to IPVAW was not supported by the data. Similarly, we found no support for the reciprocal association between tolerant attitudes and exposure to IPVAW.

The current research extends studies that have examined association between tolerant attitudes towards IPVAW and exposure to IPVAW, by providing new evidence on contextual effects on exposure to IPVAW. At community-level, we observed various patterns of association between witnessing, attitudes and exposure to the three forms of IPVAW. We found that increasing proportion of women in the community with tolerant attitudes was significantly positively associated with spousal sexual and emotional abuse, but not significantly association with spousal physical abuse. In addition, we found that increasing proportion of men in the community with tolerant attitudes and increasing proportion of women who had witnessed IPVAW in the community was significantly positively associated with spousal physical abuse, but not significantly association with spousal sexual and emotional abuse. These significant community-level factors suggest that researchers trying to study factors associated with exposure to IPVAW should consider both characteristics of individuals and where people are residing. The results of measurement models (confirmatory factor analysis) suggest that the five question scenarios on gender roles appear to be a sound tool for the assessment of tolerant attitudes towards IPVAW. There was positive correlation between all three types of IPVAW at individual-level. More importantly, the findings uncover new evidence about the correlated nature of three components of IPVAW, spousal physical, sexual and emotion abuse, at community-level. This finding corroborate those of previous studies that found that physical violence in intimate relationship almost always is accompanied by emotional abuse, and in one-third to over half of cases, by sexual abuse [28]. The findings of this study are consistent with previous studies that have examined the association between contextual factors and exposure to IPVAW [27], [29], [30]. For example, Gate and colleagues examined individual and contextual factors associated with the occurrence of IPVAW among ever-married women using the 2000 Haiti DHS and found that neighbourhood poverty [30] and high community female headship [27] was associated with increased risk of exposure to sexual violence. Similarly, Boyle and co-researchers found that women place of residence and community-level education were associated with IPVAW in India.

### Study limitations and strengths

Our study has a number of limitations that must be considered when interpreting our results. Although we compared alternative models to enable inferences about casual pathways, cross-sectional nature of the data limits ability to draw casual inferences. Though, it is mathematically possible to test reciprocal association using cross-sectional data, however, the validity of such results have been debated an questioned in the literature [31]. Since causes precede effects, prospective longitudinal studies have been suggested to be more appropriate for testing reciprocal relations [31]. Our findings on hypothesis related to witnessing IPVAW in childhood may be influenced by sampling bias. In this study, we looked at three forms of IPVAW (physical abuse, emotional abuse and sexual abuse), while study's data about witnessing IPVAW in childhood was limited to physical abuse only (“did her father ever beat her mother”). This possible sampling bias, could explain the study's findings that physical abuse is more common in communities with increasing female witnessing IPVAW in childhood. In addition, the questions used in defining attitudes towards IPVAW are may not cover all possible triggers of IPVAW. For example, questions related gender inequalities (women's education attainment, employment and financial status) were not covered [32]. Another potential threat to validity of this study is possibility of under-reporting of spousal sexual violence. We found among the couples studied; only 2% reported spousal sexual abuse. It is possible that spousal sexual abuse is under-reported among these couples. WHO multi-country study on women's health and domestic violence against women estimated the lifetime prevalence of sexual partner violence from 6% (city sites in Japan and Serbia and Montenegro) to 59% (Ethiopia province), with most sites falling between 10% and 50% [33]. There are many reasons women may under-report spousal sexual violence [34], [35]. Some of these factors were stated in Smith [36] excellent discussion of this issue, “Abuse women may not reveal her victimization to an interview for variety of reasons. She may feel that the subject is too personal to discuss, she may be embarrassed or ashamed, she may fear reprisal by her abuser should he found about the interview, she may misunderstand the question, or she may think the abuse was too minor to mention. She may even have forgotten about it. If the abuse was especially traumatic, she may not want or able to recall it”.

Limitations notwithstanding, this study makes several key contributions to the existing literature. DHS are considered to be of high quality, because they are based on proper sampling methodology with considerable high response rate and are population-based with nationwide coverage. In addition, DHS team DHS also adhere to strict ethical rules in the collection of domestic violence data. In the present investigation, more appropriate and recent multi-level structural equation modelling techniques were used to examine the association between tolerant attitudes towards IPVAW and exposure to IPVAW. Analysing hierarchical data (couples nested within communities) as individual observations (neglected clustering within the community) may lead to false positive findings, because such analysis may result in a small standard error and wrong statistically significant result [37]. In addition, we adopted a multivariate analytic framework. We considered spousal physical, sexual and emotional abuse as distinct, yet related, states nested within individuals. The multivariate analytic framework offered three distinct advantages. First, it is only through a multivariate framework that comparable assessments of common individual-level tolerant attitudes towards IPVAW that affect spousal physical, sexual and emotional abuse can be made. Secondly, the multilevel framework permits an assessment of whether local communities' tolerant attitudes make a difference to women exposure to spousal physical, sexual and emotional abuse. Thirdly, the important advantage from treating three outcomes together in a multivariate multilevel structural equation statistical framework is the estimation of the “covariance” between spousal physical, sexual and emotional abuse at the individual and community-level. In addition, this study is among first to examine the construct validity of five questions used to measures attitudes towards IPVAW using confirmatory factor analysis.

### Conclusion

Drawing upon structural equation and multilevel perspectives, in this paper we have offered an alternative to more traditional ways of thinking about the association between tolerant attitudes towards IPVAW and exposure to IPVAW at the population level. In particular, we have demonstrated that community tolerant attitudes context in which people live is associated with exposure to IPVAW even after taking into account individual tolerant attitudes. Findings highlight the importance of studying IPVAW not only in the context of individual-level factors but also within the broader community context. Future research also should address the mechanisms that connect the people and community levels, that is, the means through which contextual effects are transmitted to the individual residents. These mechanisms are crucial to the design of community-based interventions because these processes may be more amenable to change. Thus, public health interventions designed to reduce IPVAW must address people and the communities in which they live in order to be successful.
